# Tender Endothelium Syndrome: Combination of Hypotension, Bradycardia, Contrast Induced Chest Pain, and Microvascular Angina

**DOI:** 10.1155/2016/8574025

**Published:** 2016-02-14

**Authors:** Shivesh Goberdhan, Soon Kwang Chiew, Jaffer Syed

**Affiliations:** ^1^Department of Internal Medicine, Queens University, Kingston General Hospital, 76 Stuart Street, Kingston, ON, Canada K7L 2V7; ^2^Department of Cardiology, McMaster University, St. Catharines Hospital, 1200 4th Avenue, St. Catharines, ON, Canada L2S 0A9

## Abstract

Hypotension, bradycardia, and contrast induced chest pain are potential complications of cardiac catheterization and coronary angiography. Catheter-induced coronary spasm has been occasionally demonstrated, but its relationship to spontaneous coronary spasm is unclear. We describe a 64-year-old female who underwent coronary artery bypass surgery in 1998 on the basis of an angiographic diagnosis of severe left main disease, who recently presented with increasingly frequent typical angina. Repeat coronary angiography was immediately complicated by severe chest pain, hypotension, and bradycardia but demonstrated only mild disease of the left main artery and entire coronary tree with complete occlusion of her prior grafts. This reaction was almost identical to that observed during her original coronary angiogram. We now believe her original angiogram was complicated by severe catheter-induced left main spasm, with the accompanying contrast reaction attributed to left main disease, and the occlusion of coronary grafts explained by the absence of significant left main disease. The combination of these symptoms has not been documented in the literature. In this instance, these manifestations erroneously led to coronary bypass surgery. It is unknown whether routine, systematic injection of intracoronary nitroglycerin prior to angiography might blunt the severity of such reactions.

## 1. Introduction

Typical angina is defined by three features: substernal location chest discomfort, provocation by exertion or emotional stress, and relief by rest or nitroglycerin. When only two of the above criteria are met, atypical angina is suggested, while the presence of only one feature suggests noncardiac chest pain [1]. Epicardial coronary artery spasm also manifests as substernal chest pain but usually lacks a clear association with exertion and can be difficult to diagnose due to the fleeting nature of symptoms and wide range of electrocardiogram (ECG) findings, although transient ST elevation is most commonly seen [2]. Coronary microvascular dysfunction (CMVD) involves the coronary microcirculation, sparing the epicardial arteries. Microvascular angina (MVA) is a clinical manifestation of CMVD and can be seen in patients who present with anginal pain, without epicardial coronary disease [3]. Stable primary MVA refers to angina episodes related to effort without cardiac or systemic disease; but inclusive to this diagnosis are those with diabetes mellitus and uncomplicated hypertension. Risk factors for CMVD are similar to those for epicardial CAD and include dyslipidemia, diabetes mellitus, and smoking, yet the precise pathophysiology is poorly understood [3]. Coronary artery spasm is also reported in 1%–5% of percutaneous coronary interventions and can be induced via guide wire insertion. The mechanism surrounding this is believed to be a result of increased vasomotor tone and mechanical stimulation from the catheter tip [2, 4].

There are many adverse reactions that can occur during coronary angiography, involving both the catheterization process and the use of radiocontrast dye [5]. Catheter-induced vasospasm is uncommon but important to recognize and distinguish from atherothrombotic disease [6]. Hypotension and bradycardia are well known complications of coronary angiography and are directly correlated with the hyperosmolality of the contrast. Ionic contrast is associated with a greater incidence of mild to moderate adverse reactions than nonionic low-osmolar agents. These reactions include bradycardia, chest pain, transient hypotension, and elevation of left ventricular end diastolic pressure [5]. We report a case in which a patient presented with potential MVA, and, during coronary engagement with iohexol (nonionic, low osmolality contrast), the patient experienced hypotension, bradycardia, and extreme chest pain.

## 2. Case Report

A 64-year-old woman, with a history of double vessel coronary bypass surgery (CABG) in 1998, presented with five months of increasingly frequent exertional chest tightness and dyspnea. In 1998, the patient presented similarly with a few months' history of exertional chest heaviness, dyspnea, and jaw numbness. After a positive treadmill stress test demonstrating ST depressions, diagnostic coronary angiography was complicated by severe hypotension immediately upon catheter engagement of the left main artery, with blood pressure falling to less than 50 systolic and accompanied by severe chest pain. Limited angiographic images obtained demonstrated an 80% left main stenosis, with angiographically normal vessels in the remainder of the coronary tree. The patient was kept in hospital and sent for double vessel CABG, receiving a left internal thoracic artery (LITA) graft to LAD and saphenous vein graft to obtuse marginal. Since 1998, the patient had been relatively asymptomatic up until five months prior to current presentation.

Her current symptoms were similar, though not identical to her initial presentation in 1998, but they were still provoked by activity and relieved with nitroglycerin spray and rest. She also reported a significant decrease in energy and her usual activities were limited due to exertional dyspnea. Review of systems was otherwise noncontributory. Notably, she was an active user of tobacco, smoking half a pack a day for the past ten years, was a social drinker, and had a mother who died from a heart attack in her early 60s. Her medical history was significant for gastroesophageal reflux, hypertension, dyslipidemia, and hypothyroidism. Her medications included atenolol, ezetimibe, rosuvastatin, paroxetine, and l-thyroxine. She had also recently been placed on topical nitrate patch. Her new-onset symptoms prompted a referral for repeat coronary and graft angiography, and possible percutaneous intervention if appropriate. Based on her cardiovascular history and current suggestive symptoms, stress testing was decided against, due to her high pretest probability of having ischemic disease.

Prior to the procedure the patient had a benign physical examination with a resting ECG demonstrating sinus rhythm, with nonspecific T-wave inversions in V1 and V2. Access during the procedure was gained via right femoral artery where a 6-French sheath was inserted. Her baseline blood pressure was 110/70 mmHg. 6-French JL 4.0 and JR 4.0 catheters were used for selective coronary engagement. Immediately upon first injection of left coronary system with Omnipaque® (nonionic, low osmolality radiocontrast dye), she developed severe chest pain, hypotension (systolic blood pressure dropped to 80 mmHg), and bradycardia. Atropine 0.5 mg IV resulted in improvement of hemodynamics but had no impact on the severity of chest pain, which was reproduced with each contrast injection of the coronaries. Following atropine-related improvement in hemodynamics, intracoronary nitroglycerin was administered and she was able to tolerate completion of the procedure. Notably, chest pain severity was similar between injections of the right and left coronary systems. At case end, her hypotension and bradycardia had completely resolved; she was clinically pain-free and did not recall the pain during the procedure.

**Figure 1:**
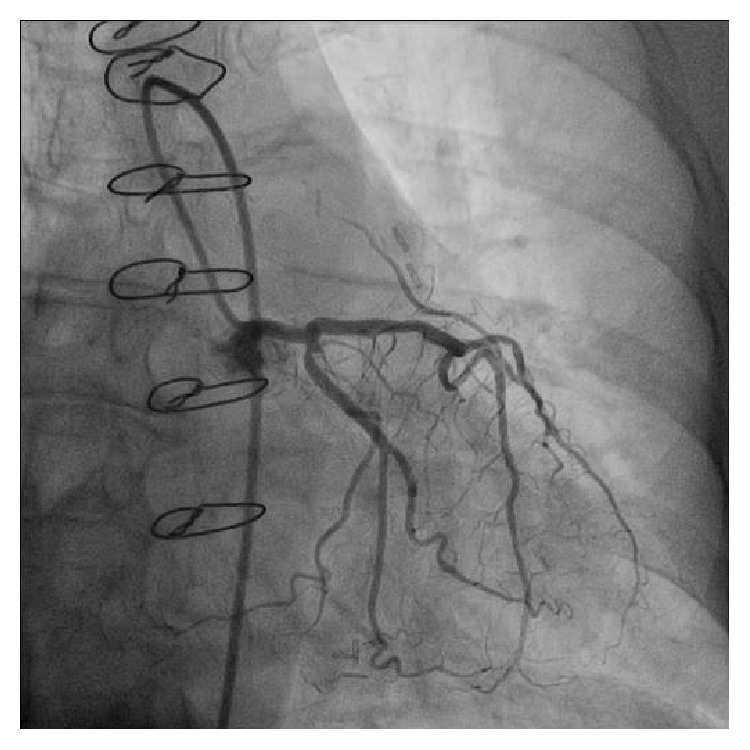
This is a selective injection of the left coronary system in the AP Caudal projection, demonstrating a large left main coronary artery free of obstructive narrowing, a mild proximal circumflex stenosis, and very minor disease of both ongoing circumflex and LAD. Retrograde filling of a small calibre LITA graft can be seen.

**Figure 2:**
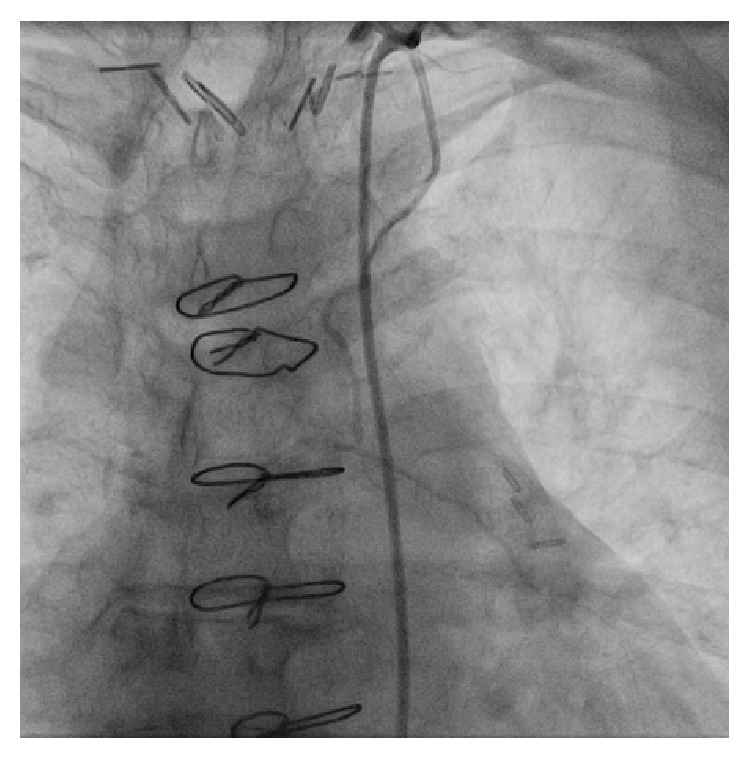
This is a selective injection of the LITA graft in the AP, demonstrating it to be of very small calibre and functionally occluded distally.

In contrast to her original catheterization procedure of 1998, selective coronary angiography failed to demonstrate evidence of hemodynamically significant stenosis within the left main coronary artery and remaining coronary tree (as shown in Figure 1). In addition, there was complete occlusion of the saphenous vein graft to the obtuse marginal (OM) and functional occlusion of the LITA graft to LAD, both of which appeared chronic (as shown in Figure 2). There was also normal left ventricular systolic function. Medical management was recommended, as well as risk factor modification, and she was strongly counseled on the importance of smoking cessation. The patient was discharged the same day and follow-up was arranged.

## 3. Discussion

This case report describes a patient presenting with typical angina without correlative angiographic findings, with unique features of procedural chest pain, bradycardia, and hypotension during selective coronary injection. These findings stand in contrast to those of her original procedure in one key respect: the absence of significant left main disease. We believe the original procedure to have been complicated by severe catheter-induced spasm of the left main artery, but this was misinterpreted as a fixed stenosis resulting in the performance of coronary artery bypass surgery. Thus, the documented occlusion coronary grafts are easily explained by the patient's lack of obstructive, atherosclerotic CAD.

A wide range of adverse effects have been described with the use of contrast media during cardiac angiography, including allergic reactions, reduced myocardial contractility, hypotension, nausea, vomiting, bronchospasm, fatal arrhythmias, pulmonary edema, and embolic events [5, 7]. During our patient's recent coronary angiography, Omnipaque, a nonionic, low-osmolar contrast, was used. When compared to high osmolar ionic media, the use of Omnipaque has been associated with significantly reduced complications [8].

Cardiac catheterization has been commonly known to cause coronary ostial spasm, most typically the right coronary artery, in contrast to the left main coronary artery [9]. Catheter-induced spasm is often related to mechanical irritation and excessive catheter torque. Patient factors regarding catheter-induced spasm include excessive vasomotor tone, early endothelial dysfunction, and active smoking [9].

Interestingly, the chest pain experienced by the patient during the recent angiogram was unlike her presenting cardiac angina symptoms. In review of the literature, chest pain labeled as mild/moderate has been noted in patients receiving iopamidol and ioxaglate, although the frequency of this symptom was low (16/500 cases between the two contrast dyes) [5]. The mechanism of chest pain related to the injection of contrast is not established. Another study documents angina as an adverse effect of iohexol, observed in 27 patients out of 1077, although the anginal events were not specifically described or compared to their presenting symptoms [7].

Based on her typical clinical symptoms and lack of atherosclerotic disease at angiography, our patient is suspected of having microvascular angina (MVA). The diagnosis of MVA could be explored further in this patient and could involve vasodilator tests, response to vasoconstrictor stimuli, and intracoronary Doppler studies but such tests have poor sensitivity and specificity and additional patient risk, and would likely not change clinical management [3]. Myocardial ischemia related to CMVD is not a well-understood phenomenon but as the abnormalities may not be uniformly distributed amongst a major coronary branch, objective evidence is difficult to obtain [3].

It was decided that our patient would be treated medically. Recommendations included discontinuing her beta-blocker, given the known propensity of such agents to worsen vasospastic phenomena, and she was aggressively counseled on the importance of smoking cessation and how this might improve her symptom control [3]. An increase in the dose of her topical nitrate and the addition of a calcium channel blocker were also discussed. While microvascular angina, catheter-induced spasm, and chest pain during coronary injection have individually been described, we believe the presence of all three features in a single patient represents a unique finding. Although routine administration of nitrates prior to angiography may not be feasible, possibly cases with left main ostial/shaft or right coronary ostial lesions could benefit from pretreatment. Systematic employment of intracoronary nitroglycerin, meticulous catheter technique, and an awareness of such issues are important for both clinicians and angiographers alike.

## 4. Conclusion

This is a unique case of a 64-year-old who erroneously underwent coronary bypass surgery after what now seems to be severe catheter-induced left main spasm. In combination with severe chest pain and hypotension with contrast injection, these symptoms together have not been seen in the literature. It is imperative to note that since the mechanisms of microvascular angina are not fully understood, it cannot be concluded as to whether all of these symptoms are connected. It is unknown whether routine, systematic injection of intracoronary nitroglycerin prior to angiography might blunt the severity of such reactions, and it is important for angiographers and clinicians to be aware of this potential combination.

## References

[1] (2012). 2012 ACCF/AHA/ACP/AATS/PCNA/SCAI/STS Guidelines for the diagnosis and management of patients with stable ischemic heart disease. *Journal of the American College of Cardiology*.

[2] Stern S., De Luna A. B. (2009). Coronary artery spasm: a 2009 update. *Circulation*.

[3] Lanza G. A., Crea F. (2010). Primary coronary microvascular dysfunction: clinical presentation, pathophysiology, and management. *Circulation*.

[4] Perera D., Patel S. J., Redwood S. R. (2003). Catheter induced spasm: a trap for the unwary. *Heart*.

[5] Gertz E. W., Wisneski J. A., Miller R. (1992). Adverse reactions of low osmolality contrast media during cardiac angiography: a prospective randomized multicenter study. *Journal of the American College of Cardiology*.

[6] Mohammed A. A., Yang A., Shao K. (2013). Patients with left main coronary artery vasospasm inadvertently undergoing coronary artery bypass grafting surgery. *Journal of the American College of Cardiology*.

[7] Matthai W. H., Kussmaul W. G., Krol J., Goin J. E., Schwartz J. S., Hirshfeld J. W. (1994). A comparison of low- with high-osmolality contrast agents in cardiac angiography. Identification of criteria for selective use. *Circulation*.

[8] Levorstad K., Vatne K., Brodahl U., Laake B., Simonsen S., Aakhus T. (1989). Safety of the nonionic contrast medium omnipaque in coronary angiography. *CardioVascular and Interventional Radiology*.

[9] Lingegowda U., Marmur J., Cavusoglu E. (2005). Catheter-induced spasm of the left main coronary artery anatomic “kinking” in its course. *Journal of Invasive Cardiology*.

